# Addiction and will

**DOI:** 10.3389/fnhum.2013.00545

**Published:** 2013-09-11

**Authors:** Brian Johnson

**Affiliations:** Department of Psychiatry, State University of New York Upstate Medical UniversitySyracuse, NY, USA

**Keywords:** neuropsychoanalysis, SEEKING, drive, psychoanalysis, addiction, toxoplasmosis, cathexis, will

## Abstract

A hypothesis about the neurobiological bases of drive, drive reduction and will in addictive illness is presented. Drive reduction seems to require both SEEKING and gratification. Will is the everyday term for our experience of drives functioning within us. Addictive drugs take over the will by altering neurotransmission in the SEEKING system. As a result of this biological change, psychological defenses are arrayed that allow partial gratification and reduce anxiety about the consequences of drug use. Repeated partial gratification of the addictive drive creates a cathexis to the drug and the drug seller. It also keeps the addicted person in a permanent state of SEEKING. The cathexis to the drug and drug seller creates a difficult situation for psychoanalytic therapists. The actively addicted patient will have one set of feelings for the analyst, and a split off set of feelings for the drug dealer. Addictive neuroses, which feature a split transference, are contrasted with Freud’s concept of transference and narcissistic neuroses. For treatment of an actively addicted patient, the treater must negotiate the split transference. By analyzing the denial system the relationship with the drug dealer ends and the hostility involved in addictive behavior enters the transference where it can be interpreted. Selling drugs that take over the will is a lucrative enterprise. The addictive drug industry, about the size of the oil and gas industry worldwide, produces many patients in need of treatment. The marketers of addictive drugs understand the psychology of inducing initial ingestion of the drugs, and of managing their addicted populations. The neuropsychoanalytic understanding of addiction might be used to create more effective public health interventions to combat this morbid and mortal illness.

## INTRODUCTION

*Toxoplasma gondii*, a single-celled protozoan parasite, manipulates the brain of the rat to shift their response to cat odor from defensive to sexual attraction (House et al., 2011). *Toxoplasma* in the environment gain entry into the rat by skin contact. They multiply in brain and muscle tissue. The immune response of the rat results in cyst formation.

The pathways for the defensive and reproductive pathways run in parallel from the olfactory bulb to the medial amygdala and hypothalamus in close anatomical proximity (House et al., 2011). *Toxoplasma* cysts elaborate tyrosine beta hydroxylase, the rate-limiting enzyme in the pathway from the tyrosine in food to dopamine. Prandovszky et al. (2011) found that infected brain cell cultures had a threefold increase in dopamine compared to uninfected cells. Alteration in brain dopamine results in a subtle shift in behavior. Rats infected with *T. gondii* shift their behavior from fearing/avoiding cats to approaching them as if they were desirable mates. The rats get eaten by the cats.

The organism makes its way into its preferred host, the cat, by taking over the brain of the rat (House et al., 2011). The rat behaves normally in all ways except that instead of avoiding cats, it seeks them out. This behavioral change is not for the survival of the rat, but for the survival of the *T. gondii* parasite.

The goal of this communication is to ask the question about whether a homologous process in the human has to do with the sale of addictive drugs. Like the process involving *T. gondii*, the mechanism would involve the dopaminergic system. The conclusion would be that, like the rat serving the *Toxoplasma* organism, the behavior of a human taking addictive drugs into their brain has to do with the benefit of the drug seller; even at the cost of the life of the addicted human.

There is almost no clinical material in this paper. Psychoanalytic and neuropsychoanalytic treatment of addiction has been extensively presented by the author in numerous clinical papers covering alcohol (Johnson, 1992, 1993, 2003a, 2011), marijuana (Jones et al., 2005), cocaine (Johnson, 2009a), heroin (Johnson, 1999, 2001, 2010), nicotine (Johnson, 2003a), and drug dreams (Johnson, 2001, 2003b, 2006, 2012) including papers describing whole psychoanalyses with 9-year follow ups after treatment for alcohol addiction (Johnson, 2011) and heroin addiction (Johnson, 2010). After 10 mostly clinical papers, this discussion focuses on the general implications of concepts derived from the confluence of neuroscience and clinical psychoanalytic work.

## DRIVE AND CATHEXIS: KEY CONCEPTS FOR ADDICTION NEUROPSYCHOANALYSIS

Drive and cathexis are concepts that originated during Freud’s pre-psychoanalytic period of neuroscience research (Compton, 1983a). Since then, the field has struggled with the problem of how to combine clinical observational data with theoretical constructs that involve a completely unconscious aspect of motivation. Freud originally identified drive as the engine of relatedness, the primary conceptual device in psychoanalysis for explaining mind, body, and environment relationships (Compton, 1983b). For some psychoanalysts, the concept of drive has taken on the extreme opposite quality. For example, Aron (1999, p. 259) stated, “…The classical drive/structure metapsychology … narrows our view of people, deprives them of subjectivity, and reduces them to objects. This limitation is true of any asocial, ‘one-person’ psychology.”

On the other hand, drive or motivation has become a focus of the neuroscience approaches to addiction. For example, Kalivas and Volkow (2005, p. 1403) stated, “As the pursuit for the neural basis of addiction advances, it is clear that the search intimately involves understanding the neurobiological basis of motivation and choice for biological reward, such as food and sex, as well as more cognitively and experientially based reward, such as friendship, family and social status.” Neuroscientists working in the field of motivation and volition have varying opinions as to the origin of motivation. Some reviews (Zhu, 2003; Haggard, 2008) look to cortical pathways as the initiators of actions. Others (Berridge, 2004; Kalivas and Volkow, 2005) focus on the dopaminergic pathway that leads from the ventral tegmental area (VTA) of the midbrain through the lateral hypothalamus to the nucleus accumbens as the central structural pathway involved in motivation. This is the SEEKING system (Panksepp, 1998).

In neuropsychoanalyst Jaak Panksepp’s model, derived from his extensive experimentation with animals, the SEEKING system has the capacity to shift activity from one to another motivational system, depending on internal and environmental inputs. This supraordinate flexibility is exactly what Berridge (2004, p.201) cited against the drive model. “The most dramatic evidence against dedicated drive neurons came from studies of motivation by electrical brain stimulation… For example, if one stimulated the lateral hypothalamus of different rats, many rats might show eating behavior. But a few rats might show drinking behavior, a few show sexual behavior, or others show predatory aggressive behavior, depending on the availability of stimuli and on the disposition of the individual rat being stimulated.” This phenomenon, that various motivated behaviors are prompted by one system, is exactly the concept of drive; a constant pressure from inside the organism to do work (Freud, 1915; Shevrin, 1997; 2003).

In an earlier contribution (Johnson, 2008) I considered addiction researchers Robinson and Berridge (1993), Berridge and Robinson (2003) distinction between “wanting” and “liking.” In that paper I posited that Panksepp’s SEEKING system is the same system that Freud (1915) hypothesized to exist deep in the brain, libidinal drive. My assumption was not based on a historical study. I was and am using Freud’s thinking only because, in many cases, it is still the most perspicacious description integrating nomothetic neuroscience with ideographic psychoanalytic observation. I suggested that Freud’s concept of the pleasure principle was connected to Panksepp’s many observations of endorphin/opioid function in the brain (Panksepp, 1981, 1990, 1998, 1999; Panksepp and Watt, 2011, etc.). Importantly, the pleasure system is tied to the drive system via opioid receptors in the VTA and nucleus accumbens shell (NAS), where they potentiate glutamatergic and dopaminergic processes that intensify drive.

Using the SEEKING system to stand in for Freud’s drive system obviates the objection that it excludes a focus on relatedness, since SEEKING and other instinctual systems, CARE, PLAY, LUST, and PANIC (Panksepp, 1998), offer a biologically based and much more articulated set of instinctual drivers toward forming relationships. It gives us a model for clinical work that preserves Freud’s concept of libidinal drive while responding to Aron’s concern that drive produces an “asocial, one person psychology.” We SEEK relationships. Panksepp’s neuropsychoanalytic formulation of instinct, which is based in animal research, solves problems which could not be understood by an approach that is purely based on clinical experiences with patients.

Freud described “cathexis” as an initially mobile instinctual energy that could be bound to persons, body parts, ideas, or dream elements (Freud, 1920, p.34). In the 21st century we have information that allows us to describe the neuroscience of libidinal investment (Johnson, 2008). Dopamine is released in the nucleus accumbens of mother rats following pup exposure. VTA or nucleus accumbens (drive/SEEKING system) lesions disrupt maternal behavior (Insel, 2003). Dopamine (D1) receptors are necessary for rats to develop place conditioning for opioids. Opioid receptor antagonists block the development of partner preference in rats after mating. Both drive and pleasure need to be functioning to produce attachment. Without the drive of the dopaminergic system, there can be no libidinal investment. In order to form a sexual bond, rats have to remember that mating was a pleasant, not just a driven, experience.

Other neural systems are involved when animals come to prefer specific mates. Medial orbital frontal, amygdalar, and hippocampal memory inputs are involved. Hormonal systems interact with drive and pleasure systems. Oxytocin potentiates endorphin release during mating. Oxytocin is essential for partner preference, as has been demonstrated repeatedly in experiments comparing the prairie vole, which has an oxytocin system, with the montane vole, which does not (Insel, 2003; Johnson, 2008). Prairie voles form sexual partnerships, montane voles do not. In summary, cathexis has to do with an ensemble of drive, pleasure, memory, hormones – but there is no cathexis without drive.

One conclusion of the 2008 paper was that versions of the 1993 Robinson and Berridge distinction between wanting and liking had already been discovered by previous investigators. Freud had described it in 1920 in his essay, “Beyond the Pleasure Principle.” Panksepp had shown the distinction between wanting and liking in his papers on the SEEKING and endorphin systems (Panksepp, 1981, 1990, 1998; Panksepp and Watt, 2011, etc.). But the main conclusion of the paper was that in biological or “physical” (Johnson, 1999, 2003b) addiction, addictive drugs had changed the drive system so that they were urgently wanted; whether intoxication was pleasant or not. This conclusion is an elaboration of the concept that is generally accepted in the neuroscience community that addiction begins with an alteration in the mesolimbic dopamine system (Hyman et al., 2006; Koob and Volkow, 2010). Addiction represents the usurpation of neural processes that underlie pursuit of food, water, sex, and relationships. Implicit was an idea about cathexis that will be developed below; cathexis for drugs or drug sellers can complete with cathexis for people who are loved.

## DRIVE REDUCTION AND DRIVE

Why is the concept of drive reduction dead? I believe it is because Freud’s thinking about this topic has been ignored by current neuroscientists. For example, when Berridge (2004) discussed why drive reduction had been disproved by animal experiments, he explained that animals who only SEEK will do it forever, and animals who are only gratified, for example by having caloric requirements satisfied by gastric feeding, were still motivated to pursue food. An important source of his confusion is the behaviorist term “reward,” which conflates the concepts of SEEKING/drive and gratification. It is only by separating these two components of drive reduction that we can understand how they operate.

Freud gave the fullest description of drive reduction in his 1923 paper, “The Ego and the Id” (Freud, 1923, pp. 21–23).

“Internal perceptions yield sensations of processes arising in the most diverse and certainly also the deepest strata of the mental apparatus…they are more primordial, more elementary than sensations arising externally…

Sensations of a pleasurable nature do not have anything inherently impelling about them, whereas unpleasurable ones have it in the highest degree. The latter impel toward change, toward discharge, and that is why we interpret unpleasure as implying a heightening and pleasure a lowering of energetic cathexis…

This something behaves like a repressed impulse. It can exert driving force without the ego noticing the compulsion… Not until there is a resistance to the compulsion, a hold-up in the discharge reaction, does the ‘something’ at once become conscious as unpleasure…

The part played by word-presentations now becomes perfectly clear. By their interposition internal thought-processes are made into perceptions… We are all ‘lived’ by unknown and uncontrollable forces.”

The ego’s relationship with the id, “Is like a man on horseback, who has to hold in check the superior strength of the horse… Often a rider, if he is not to be parted from the horse, is obliged to guide it where it wants to go.”

Drive reduction involves a combination of the rapacious, insistent drive building to a state where it demands satisfaction, and the pleasure of complete gratification. Both must operate sequentially for the drive to be reduced. Drive alone, in the case of addiction the constant drive for addictive drugs, is only increased by exposure to drugs. Once addicted, exposure to alcohol, cocaine, nicotine, or opioids causes a brief diminution of desire, followed by an increase of the urgent wish for the drug.

In the case of self-stimulation of the drive center (lateral hypothalamus), animals wired to be able to activate this area will push the “on” button constantly until they die. The unpleasure of briefly relieved drive apparently causes endless fruitless attempts to achieve drive reduction. In contrast, pure gratification without activation of SEEKING is in the end unsatisfying. Pornography addiction may be an example of gratification that is endlessly unsatisfying. Masturbation may be unsatisfying because it gratifies sexually without engaging the SEEKING system. Apparently, animals or humans who can’t reduce drive by combining SEEKING and satisfaction sometimes endlessly engage in activities that activate only one or the other half of drive reduction.

We have taken as a hypothesis that drive involves activation of the ventral tegmental dopaminergic SEEKING system running from the midbrain through the lateral hypothalamus to the nucleus accumbens. A second hypothesis is that drive reduction requires both activation of the SEEKING system and gratification; food, sex, a relationship, something that requires work (Freud, 1915; Shevrin, 1997), and involves the complete relaxation of gratification. Addictive behaviors cause a brief and incomplete reduction of drive that result in endless drug seeking. The Freudian concept of will is necessary to understand how drive and drive reduction operate in the individual to change behavior.

## THE CONCEPT OF WILL

In the Project for a Scientific Psychology (aka “Psychology for Neurologists”) Freud explained, “…in the interior…there arises the impulsion which sustains all psychical activity. We know this power as the *will *– the derivative of the drives” (quoted/translated by Schmidt-Hellerau, 2001, p. 61). The experience of drives operating inside us can impel us to do things that we do not consciously “want” to do. This often leads to an interpretation by the analyst that certain behaviors are, “intentional but not conscious.” The reasons that we do things are often not apparent to us.

In fact, the lay term “will power” sometimes seems to exist as a denial of true intention. People will say things like, “I wanted to eat the whole pint of ice cream, but I exercised will power and only had half.” Or even less consciously, “I really wanted to stay on my diet and I struggled not to eat all that ice cream, but I did it anyway; I ate that ice cream against my will.” Much of the work of psychoanalysis has to do with patients becoming conscious of their real motives, what the true goals of their will is (Wheelis, 1956; The philosophical and psychoanalytic intersection of will and drive was reviewed in Young and Brook, 1994).

As Freud said so presciently, it is the repeated stimulation of neuronal pathways that leads to cathexis (Schmidt-Hellerau, 2001, pp. 54–58). The process of facilitation of neuronal pathways, leading to structural changes in the brain, would nowadays be referred to as long-term potentiation (Kandel, 2006). The combination of experience and brain changes under the influence of neurotransmitters, neuropeptides, and hormones leads to the development of interests that are different for every person (Johnson, 2008). In other words, inborn givens take shape and definition through the interaction with the social environment and establish patterns of maintaining contact and relatedness with others. This phenomenon of modulation of cathexis by a combination of innate biology, development, and experience means that every person is a little different in their tastes. The urgent needs generated by the drive system, once specific tastes in object and patterns of relatedness become fixed, are matched up against external reality. The degree of pleasure and fulfillment resulting from actualization of one’s drives and cathexes lead to either gratification or neurotic frustration (Johnson, 2008). In the case where expression of will leads to frustration, individuals feel that their life is not going well and yet are often not be able to articulate where the problem lies.

Interpretation of conflicts between conscious and unconscious goals, often described as “neurotic conflict,” is the constant occupation of any psychoanalyst. Meissner (2005, p. 28) discussed “intrasystemic id conflicts” involving motivational systems. One might love one’s mother consciously, and yet also wish to destroy her unconsciously. Both urges could be described as id-driven. This conflict generates anxiety – a signal that there is trouble in a relationship (Freud, 1926; Watt and Panksepp, 2009). There is a conflict between the wish for additive drugs and the wish for relationships. Both could be characterized as id-driven.

In a typically parallel neuropsychoanalytic way, the intrasystemic id conflict of addiction can be seen psychologically and also neurobiologically. Psychologically, one might love one’s mother consciously, and also unconsciously want to destroy her by destroying oneself with heroin. The use of addictive drugs can be understood as an unconscious expression of rage (Dodes, 1990). Neurobiologically, the conflict has to do with a conflict between urgently wanting a drug, and still wanting other goals of the drive system including a relationship with one’s mother. The experience that the mothers of heroin users are wildly upset while the heroin user sees themselves is single-mindedly pursuing drug use, is an everyday experience on an addiction treatment service.

## HOW THE WILL IS TAKEN OVER BY ADDICTIVE CHEMICALS

There are only about 20 chemicals known to humans that alter the drive system so as to create a new drive (Johnson, 2008). These substances: alcohol, nicotine, benzodiazepines, opioids, stimulants, marijuana, phencyclidine, etc., all work by diverse mechanisms (Nestler, 2005), but with the same uniform end result. They cause sensitization of the ventral tegmental dopaminergic SEEKING system to the chemical (Robinson and Berridge, 1993). After sufficient exposure to the chemical, the person begins to want the drug; irrationally and insistently.

**FIGURE 1:**
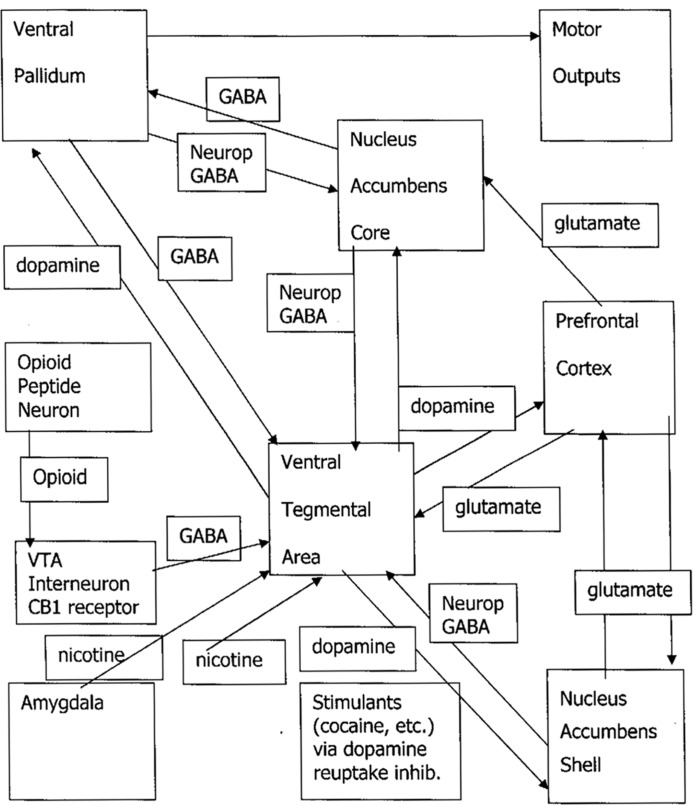
**Craving/dreaming pathways: Neurop, neuropeptides; GABA, gamma amino butyric acid, CB, cannabinoid**.

There is a triangle on the lower right half of **Figure 1** which shows the hypothesized mechanism of physical addiction for stimulant drugs. The corners of the triangle are the VTA, the NAS, and the prefrontal cortex. Cocaine and methamphetamine increase dopaminergic neural activation from the VTA directly to the NAS (Niehaus et al., 2009) by blocking the dopamine reuptake transporter protein. Nicotine has receptors on the VTA, and stimulates activating signaling from the amygdala (Nestler, 2005).

There is a tonic brake on the VTA created by GABAergic inhibition by a set of interneurons. This set of interneurons is represented by a box directly to the left of the VTA box that occupies the center of **Figure 1**. Opioids act as a brake on an inhibitory system involving GABAergic interneurons that slow dopamine neurotransmission from the VTA to the NAS (Nestler, 2005). Removing this inhibition from the VTA results in increased dopamine activation of the NAS. Marijuana’s tetrahydrocannabinol lodges in endocannabinoid receptors in inhibitory GABAergic VTA interneurons, inhibiting this brake so that there is increased dopaminergic stimulation of the NAS (Fattore et al., 2008). The mechanism for alcohol and benzodiazepines may be that in withdrawal from these GABAergic drugs, there is a lessening of GABAergic inhibition of the VTA, and dopamine neurotransmission is increased (Enoch, 2008).

The mechanism of physical addiction for every addictive drug is that dopamine neurotransmission from the VTA to the NAS is altered. Craving, the psychological manifestation of dopaminergic drive activity in this pathway (Shevrin, 1997), is induced by drug exposure. After repeated exposure to the addictive chemicals that produce dopaminergic activation of the SEEKING system, the chemicals become wanted, desired, craved; just like so called, “natural reinforcers,” food, water, sex, and relationships.

The pathway does not end with the NAS. As seen in **Figure 1**, there are limbic and frontal centers connected with this subcortical pathway. As the effects of stimulation in the subcortical pathway cause long-term potentiation of higher centers, and drugs are wanted, drug cues recognized at pathways involving amygdalar, hippocampal, and frontal activation provoke neural firing, and downgoing glutamatergic pathways increase craving by stimulating more dopamine release. The higher centers notice possible availability of drugs, and turn up craving.

The pathways in **Figure 1** allow for the concept that there are two mechanisms of induction of craving; the “upper” and the “downer” pathways. The terms “upper” and “downer” are street language for whether the user experiences a drug as activating or relaxing. These terms may also reflect a difference in neurobiological action. The upper pathway, activated by cocaine, methamphetamine, and nicotine, directly increases firing from the VTA to the NA. The downer pathway is less direct. The interneuron braking system is deactivated, leading to increased activation of VTA to NAS dopamine. This would account for the fact that the upper drugs cause drug craving so commonly and are harder to become abstinent from, while downer drugs such as marijuana, alcohol, opioids, or benzodiazepines provoke addiction with lower frequency. When drugs in the downer group are used for recreational or medical reasons, most users do not become addicted.

Persons whose brains have been changed by addictive drugs must obtain the chemical or they are punished by ferocious unpleasure if there is, “A resistance to the compulsion, a hold-up in the discharge reaction.” These chemicals are used to take over the will of the victim. For example, one might say that the person who is smoking a cigarette while having fantasies of dying of cancer, heart disease, or emphysema is not following their own will, but actually enacting the desire of the cigarette manufacturer, who is selling the cigarettes.

We might say that human children, who begin using nicotine on average in the United States at age 13, are controlled by a process homologous to rats infected by toxoplasmosis. Their behavior has been subtly altered by a change in dopamine/SEEKING. They behave in almost every way as if they are themselves. But inhaling nicotine, along with particles that can produce cancer or ruin the lung’s ability to obtain oxygen, is in the service of the cigarette sellers.

## DRUG-INDUCED RELATIONSHIPS

By using these drugs, individuals begin to “want” them for no reason other than brain changes. By associative learning, the purveyors of the drugs are also wanted. The state of drug craving is intensely emotional, urgent, energetic, searching for a means of gratification. By providing the drug, the seller becomes wanted, cathected.

Relationships with drug providers can have a yearning, romantic cast. As one patient told me, despite being in treatment for addiction, and sober, “I love my dealer!” A patient with attention deficit hyperactivity disorder (ADHD), cocaine and nicotine addiction suspected I would profit from having him buy a prescription for the antidepressant bupropion, used to treat ADHD and nicotine dependence. This suggests a transference from his drug sellers to me, with their/my drug as the intermediary causing a cathexis. A third patient, early in his treatment for opioid addiction, called the drug dealer to whom he had paid $200,000, his “best friend.” When in emotional distress, my patient refused to call my cell phone, but rather, kept relapsing because he would call his drug dealer’s cell phone.

There are secondary changes in the brain as addiction progresses. Later brain changes involve routinization of drug SEEKING by reorganization of pathways (Koob and Volkow, 2010) involving the nucleus accumbens core (Everitt and Robbins, 2005; Kalivas and Volkow, 2005) and diminished prefrontal inhibition (Bechara, 2005), especially if there are losses of brain tissue due to the various degrading effects of these drugs (reviewed in Johnson, 2009b). The longer addiction goes on, the harder it is to recover. Initiating brain changes with one drug results in faster adaptation with craving for a second addictive drug (Robinson and Berridge, 2000). For example, individuals who start smoking cigarettes before the age of 15 are 80 times more likely to use illicit drugs (Lai et al., 2000).

Exposure to addictive drugs can cause brain changes that result in permanent craving. This makes perfect sense if you think of the reason that drives are built into animals. We need to have a steady pressure to obtain items in the environment that are related to survival. If we learn where these items are, or how to recognize their possible availability, we need to have our craving turned on at that moment when we recognize availability cues so that we intensify our search for the proximal, life-supporting item, no matter how long ago we learned about the linkage between cue and drive goal. After learning about the constant availability of reward in the environment, SEEKING them can become a more automatic, unconscious behavior – modulated in the nucleus accumbens core.

However, this survival mechanism has its drawbacks. One Freudian discovery was that this constant pressure to act can come into conflict with other considerations that have to do with living in a social environment. The original paradigm of the Oedipus Complex was the conflict between the sexual drive and the presence of a larger, older, same-gender parent who was in the way of the child’s closeness with the parent of the opposite gender (Freud, 1909).

Conflict is also generated regarding drug-seeking. Once addictive drugs get entrained into the drive pathway, there is a constant pressure to act to procure the drug experience again, no matter how unpleasant and dysfunctional the consequences. This is no different conceptually than yearning for a parent of the opposite gender when one is too young to effectively or safely compete. Lust can be dangerous; whether for love or drugs. Life provokes internal conflict.

Drives are so deeply unconscious that it is hard to experience them directly. Describing “craving” is a difficult task (reviewed in Johnson, 2012). Craving seems absent 1 min, overwhelming the next. An unconscious basal state may be altered by the provocation of dopamine neurotransmission in the VTA/nucleus accumbens pathway when drug cues activate frontal or limbic centers. With a drug cue or intense emotion, the previously unconscious drive enters awareness.

Finally, just as food, water, sex, and relational needs provoke dreams, so does the hunger for drugs (Johnson, 2001). Drug dreams are a unique aspect of physical addiction (Johnson, 2003b). Craving for drugs that is not conscious can be made conscious by the interpretation of dreams (Johnson, 2001).

## PSYCHOLOGICAL SEQUELAE OF PHYSICAL ADDICTION

As soon as the drug has had sufficient impact on the neural pathways shown in **Figure 1**, there is a reorganization of thinking. We are in a position to actually see the impact of physical factors in the brain on psychology by talking to persons who have undergone this change. The victim of this process begins to have the experience described above that they become uncomfortable as the drug washes out of their brain. The addicted person has that inner sensation that they absolutely must have more of the drug to ward off the unpleasure of craving. They suffer from endless drug SEEKING. Persons with alcohol dependence show up at the bar at 8 am. Persons with heroin dependence knock on their dealer’s door early in the morning. Cigarette smokers get off planes and have a cigarette before they do anything else. The unpleasure of craving is so intense that addicted individuals do everything they can to get rid of it. But it always comes right back.

Defenses are arrayed by the ego to modulate the new drive state. Consistent with Freud’s metaphor of horse and rider (quoted in the drive reduction and drive section), the newly addicted person urgently wants to procure and use their drug. Their ego is aware of the potential liabilities in using it again. The ego deploys a series of explanations about why using the drug again is, “OK.”

Psychological defenses reduce anxiety at the expense of obscuring reality. It is customary in the field of addiction to talk about “denial.” However, there is no reason that a single defense, denial, would be arrayed against a drive. There are as many defenses arrayed against the drive for addictive chemicals as there are against any other drive. For example, an addicted person might use projection of responsibility, “I’m using because she/he treated me badly.” The defense could be minimization, “Going to work late because I was hung over from drinking isn’t such a big deal.”

Each person’s denial system is their own unique set of explanations about why they should keep using their addictive drug(s). To the outside observer, their denial system, the particular set of psychological defenses set up to protect continued dangerous drug use, makes no sense. This is because the defenses are arrayed against internal stimuli. The observer cannot feel the drive to use the drug. In fact, one of the problems of identifying with addicted individuals is that the observer or psychoanalyst usually has not had the patient’s experience of pursuing drugs. The best way to make an empathic identification is to imagine one of the basic drive goals, such as food. One can think about how hard it is to lose weight by tolerating the urge to eat things that are off the weight-loss diet. One will notice that defenses are arrayed to protect eating foods that are not consistent with the diet, just as addicted persons have defenses about using when they also wish to be abstinent.

The denial system of the physically addicted person is based on their craving for the drug, and on their allegiance to the seller of their drug. This may sound like an odd claim; isn’t the drug the object? The answer to this question puts us back where Freud started. Human relationships are grounded in the gratification of drives. In adult sexual relationships, attraction leads to increasing involvement, sexual gratification, cathexis, and loyalty.

We can understand how drive precedes cathexis in relationships if we observe the SEEKING system functioning with the drive for addictive drugs. Persons who become addicted develop an allegiance to the seller of the drug. Cigarette manufacturers are keenly aware of cathexis. Their goal is to make the user of cigarettes fall in love with the brand that contains their nicotine.

In the United States, about 20 million persons buy illegal drugs, and it is almost unheard of for an addicted person to turn in their drug dealer. In part this is because the drug users fear that they will be killed if they alert police that their dealer is selling drugs. But mostly it comes from cathexis. Addicted persons have warm feelings toward their dealers; even as they may also fear being killed by them. Warm feelings that cover a fear of being killed is the defense, “idealization.”

Craving provokes idealization. Idealization of the drug is a constant and indispensable part of denial. This defense, as described by Klein (1957), involves fear of the object (drug/drug seller). Addicted people are terrified by their behaviors. Yet this information is unavailable to them consciously. Their conscious experience is that, whatever the drug, its use is wonderful. People who smoke cigarettes are staying slim, being free to defy authority, expressing their emotions, and their sexuality – just like cigarette smoking actors do in the movies. Some persons addicted to alcohol pride themselves on how much they can drink. Some users of heroin feel that it is a cooler drug than any other. For addictive drugs that are legal, advertising has the theme of ideal behavior; that the drug or alcohol is connected with social dominance or pleasure in sports or relationships. I summarize various aspects of this idealization in **Table 1** (explained at length in Johnson, 1993, 1998).

**Table 1:** Addictive idealization/splitting.

The addiction	
Experiencing ego/psychic reality	Observing ego/external reality
Facilitates relationships	Makes close relationships difficult (wards off fear of control/merger)
Creates pleasure	Creates pain
Gives a sense of omnipotence	Makes one impaired
Is a rebellion that creates a feeling of separateness	Is a compliance with the attacking introject that undercuts the use of aggression needed to be separate

Idealization, like laughter, is catching. This may be an underlying dynamic of the social nature of the spread of cigarette smoking (Christakis and Fowler, 2008). When a 12-year-old child sees a 16-year-old child smoking a cigarette, the 16-year-old is using idealization internally to defend against their panic about seeing how out of control their behavior is and to defend against their perception of physical changes such as cough and shortness of breath. This idealization is represented to the 12-year-old interpersonally. The 16-year-old idealizes their ability to smoke a cigarette without having to cough when the irritating smoke enters their lungs, an aspect of tolerance. They communicate to the 12-year-old that smoking is appealing, “cool.” As a result, the 12-year-old victim will tolerate the aversive aspects of smoking until tolerance, craving, and denial set in. This victim is then in a position to pass the addiction on to another young victim. Other drugs work by the same mechanism. It doesn’t matter what the chemical is, the defense of idealization is uniform for addictive drugs.

## TREATMENT IMPLICATIONS OF THE NEUROPSYCHOANALYTIC CONCEPTS OF DRIVE, DRIVE REDUCTION, AND WILL

Freud had a set of conditions that he felt had to do with the distribution of libido by the ego. In the “transference neuroses” libido was available to be cathected to the analyst. This was ideal for psychoanalytic treatment. In the “narcissistic neuroses” (Freud, 1917, pp. 420–423) libido was withdrawn from objects, therefore from the analyst, and psychoanalytic treatment was impossible. As a hypothesis, we could add to this list “addictive neuroses.” Some libido is cathected to the drug/drug seller, some to other people – including the analyst. There is a splitting of the transference, just as there is a splitting of the patient’s experience (**Table 1**). What the analyst observes is that the patient has many ordinary dynamic interactions in the hour, but keeps the addictive urges outside the hour.

The patient does with the analyst (of course) what they do with all relationships. The patient very much wants to be engaged with the analyst, but has another cathexis for her/his libido that has nothing to do with the analyst. The patient’s conscious experience is that behaviors having to do with obtaining and using drugs have little to do with other relationships. Their libidinal investment is dissociated into the part that cares deeply about the analyst and the part that cares deeply about obtaining and using drugs.

One result of this situation is the familiar complaint of some addicted individuals who claim that their psychoanalysis did nothing to change their addiction. This is because the patient felt that their addiction had nothing to do with their analyst (their true experience) and their analyst was not in a position to hear about the effects of the addictive drug. In these psychoanalyses, the patient and analyst worked on their relationship, while the relationship with the drug/drug dealer remained unexamined. The unintended result of this approach can be that the patient who has completed such a psychoanalysis is even more adept at having relationships with people while using their drug addictively. Lying midway between the transference neuroses and the narcissistic neuroses, the addictive neuroses require some alterations of technique in order for the patient to benefit from treatment.

What can an analyst do when faced with an addictive neurosis that has a split cathexis/transference? The answer has something to do with developing conscious conflict about drug use within the relationship with the analyst. The patient knows that they are in trouble because of their relationship with the drug and with the drug dealer, but not consciously. The patient knows that they cannot both fully engage in the relationship with the analyst, and stay involved with the drug/drug dealer, but not consciously. The relationship with the drug/drug dealer is based on a system of beliefs which make perfect sense to the patient because they exist to diminish the anxiety about using a drug that is creating damage and may result in death. For example, many persons who are addicted to nicotine will say things like, “Sure cigarettes may kill me. We all have to die some time!”

Therefore, in many cases the analyst will have to divide the treatment into two phases. In the first, the transference is not explored because it is split. The analyst appreciates that attacking the relationship with the drug/drug dealer is not going to work. A strong cathexis has been established after many experiences of great unpleasure relieved by drugs and/or alcohol. The analyst limits their interventions to clarifications and confrontations that intensify conscious conflict between the wish to use and the symptoms of addiction that ensue from use. By using these non-transference interpretations, the analyst works on the denial system. It is only after the patient has moved through the “stages of change” by virtue of increasing dismay about the consequences of drug and/or alcohol use, and stopped using, that the treatment enters the second phase.

Addicted persons are like children in a divorce who don’t want to tell one parent what is going on with the other. They feel an alliance with both, but understand that the allegiance to one is essentially disloyal to the other. Just as one divorced parent often does not hear what the child is doing with the other, the analyst often does not hear what is going on with the drug dealer. Not being open and honest is the result. This formulation allows the treating clinician to shift from, “My patient lied to me,” to, “I encountered a split transference.” The first reaction might produce anger, the second interest, and a feeling of a technical challenge.

For any psychotherapy treatment, a key ingredient of healing has to do with the therapeutic alliance (Nissen-Lie et al., 2010). If addiction therapists use non-specific or intuitive interventions that are warmly related, outcomes might be the same despite different theoretical orientations. Therapists who intuit the underlying neuropsychodynamics more accurately might have better outcomes, but be unable to explain why. For the therapist who was able to explain what they were doing with neuroscience and metapsychology, there might be better outcomes – but this hypothesis has not been empirically tested.

An innovative psychoanalytic treatment of alcohol use disorders in borderline personality disorder, Dynamic Deconstructive Psychotherapy, subjects showed a significant decrease in heavy drinking accompanied by complete cessation of other drug use. In contrast, subjects in optimal community care received more treatment but showed increased drinking and increased drug use over the 30 month post-treatment follow up study (Gregory et al., 2010). This kind of naturalistic comparison of outcomes for patients who are initially randomized into neuropsychoanalytic or conventional treatment groups would be a way to empirically test the concepts described here.

Talking with patients during active addiction, one becomes aware that use is procedural, automatic, unconsidered. The technique of the first phase of neuropsychoanalytic treatment of addiction is to sharpen the conscious conflict between the drive derivative that is in evidence during use, but not conscious, and the ego’s alarm at the reality of the consequences. Caring is communicated. Denial is undercut. We must remember the earlier quote from Freud regarding the function of word-representation as the mechanism by which internal unconscious thought processes are made into perceptions. In order for the addicted person to continue to be actively addicted, they can’t think about what they are doing. Talking about one’s urge to use drugs and alcohol takes place within a relationship. As Freud said about word presentation, “It is like the theorem that all knowledge has its origin in external perception.… A hypercathexis of the process of thinking takes place, thoughts are *actually* perceived – as if they came from without – and are consequently held to be true” (Freud, 1923, p. 23). Talking about craving and addictive behaviors changes them from precontemplative unformulated experience to more conscious problems that require work in psychotherapy.

If the goal of the ego is to serve the id, like the rider guiding the horse where it wants to go, then the patient will resist talking about their addiction because it disrupts the ability to go get drugs. In this way, the powerful urges created by exposure to addictive chemicals debilitate ego functioning. This impairment is often experienced by the analyst as having a patient who says, “Nothing comes to mind,” or who does not arrive for treatment. Recognition of this injury to ego functioning by an altered drive state can be ameliorated by interpreting the lack of association or the missing of appointments as manifestations of craving. For example, the analyst may respond to a patient who says, “Nothing comes to mind,” with, “Perhaps you are thinking about using drugs, and you are trying NOT to talk to me about that.”

The psychoanalyst should not take idealization at face value. A patient who romances their addictive behavior can be listened to until the negative/frightened side of the thinking emerges. The alternative to addictive idealization is conscious ambivalence.

What happens when the denial system is interpreted sufficiently so that the patient stops using? My observation is that the split transference collapses, and the issues that had been diverted into addictive drug use enter the transference. I reported, “The psychoanalysis of a man with active alcoholism,” where the end of alcoholic drinking during days per week psychoanalysis resulted in intense hostility entering the transference (Johnson, 1992). In the second phase of treatment, aggressive derivatives (Dodes, 1990) that had been expressed through the use of alcohol entered the transference relationship, where they were explored and ameliorated (Johnson, 1992). For some patients, when addictive behavior stops, the analyst has to be prepared for a siege of hostility that had never been in evidence during the first phase of treatment. The addictive behaviors had expressed the hostility and displaced it away from the transference.

I reported the 4 days per week psychoanalysis of a man with heroin addiction where cessation of heroin use resulted in an intense anaclitic depression entering the transference (Johnson, 2010). The patient went from a cool, unrelated person to an intensely needy and frightened person. The amelioration of the anaclitic depression within the transference resulted in a 9-year absence of addictive symptoms at the time of the report.

## PUBLIC HEALTH APPLICATIONS OF THE NEUROPSYCHOANLYTIC MODEL

Psychoanalysis has been since its early days a theory of culture as well as of the individual mind (Paul, 2011). The discussion so far has described an illness that is based in a brain system that is deeply unconscious. Items that impinge on the drive pathway are “needed.” Addictive drugs are a commodity. The property of these drugs, that they take over the will by creating dysphoria/craving/unpleasure during abstinence, means that addicted persons will do just about anything to obtain their drug.

How widespread is addiction, and how much money is involved in addiction? Let’s think about the number of brains involved. 26% (World Health Organization [WHO], 2010) of the world’s population of almost seven billion smokes cigarettes. This amounts to about 600 billion cigarettes/year sold (World Health Organization [WHO], 2008). 13% of the world’s population drinks at least 40 g of alcohol (three drinks) per day (World Health Organization [WHO], 2010). There are nearly two billion people using nicotine and one billion people drinking substantial amounts of alcohol.

The amount of money involved in selling legal drugs seems not to be carefully tracked worldwide. We know facts such as Philip Morris International was the 14th most profitable company in the United States in 2008, making $6.89 billion in profits (CNNMoney.com 2010). The United Nations Office on Drugs and Crime (United Nations Development Programme, 1999; Reuter et al., 2009, p. 3) estimates that the illicit drug industry accounts for 8% of world trade, about the same size as the oil and gas industry or world tourism. Drugs that impinge on the SEEKING system have many customers.

How lethal and morbid are drugs and alcohol? Worldwide tobacco accounts for 9% of all deaths, 18% in high income (>$10,000 US) countries (World Health Organization [WHO], 2010). Alcohol causes 4% of deaths worldwide, 2% in high income countries (World Health Organization [WHO], 2010). Alcohol is the #3 leading global risk for burden of disease behind starvation and unsafe sex, and tobacco is #5 (World Health Organization [WHO], 2010). Cigarettes kill about half of the persons who use them (World Health Organization [WHO], 2008), which adds up to 443,000/year in the United States (Centers for Disease Control [CDC], 2009) and about four million/year in the world (World Health Organization [WHO], 2008).

How would we account for this apparently “irrational” economic activity? The SEEKING system of cigarette smokers has been captured by nicotine. If a seller can induce a potential victim to expose their brain to an addictive drug a few times, the alteration in the drive pathway will make the person want the addictive drug despite the danger. For this reason, purveyors of addictive drugs use a sophisticated psychological understanding of idealization and splitting to attract and manage their customers. For example, the nephew of Sigmund Freud, Edward Bernays, and one of the founders of American psychoanalysis, A. A. Brill, went to work for American tobacco companies in the 1920s to help with campaigns to attract new populations to the smoking of cigarettes (Brandt, 2007).

Many smokers say that they don’t want to smoke, even though they show that they do want to smoke. Whose will are they following? The addicted person is following the will of the seller. While the smoker knows they cannot possibly benefit from their addictive behavior, the entire industry of production, marketing, and sales benefits enormously. This is true of any industry that produces a chemical that becomes urgently wanted by altering the drive pathway; whether a government deems that chemical legal or illegal.

We may be attracted to many people, but we make relationships based on cathexis. We fall in love with people who can meet our needs; conscious or otherwise. Love is irrational. The addictive drug industry is successful by capturing the will and the cathexis of its victims.

This information is of value in combating addiction. Public health initiatives informed by concepts such as the capture of will and cathexis, idealization of drug use, and the financial consequences of having a commodity with these properties, would lead to much different behavior by governments. For example, the addictive drug industry might be nationalized to divert money away from those who profit by deceptive advertising to teenagers, and to properly inform the public about how addictive drugs work in the brain to produce bizarre behaviors. It is more desirable from a harm reduction standpoint to have heroin sold in state stores by drug counselors than by gangs on the street with guns. It is more desirable from a harm reduction standpoint to have methamphetamine profits go to government revenues than drug cartels.

Finally, returning to the idea of the homology of the way that *T. gondii* controls the brain of the rat, and the way that protagonists of the addictive drug industry control the brain of the addicted customer, we notice that in both cases there are examples of random collateral damage. Toxoplasmosis is an important human disease, affecting about 1/3 humans in the world (House et al., 2011). There is no particular advantage for the *Toxoplasma* to inhabit the human brain since the organism dies there when the human dies.

There is speculation that the parasite expresses dopamine in the human brain, producing in some hosts schizophrenia or obsessive compulsive disorder (House et al., 2011). The dopamine blocker haloperidol moves the behavior of both humans and rats back toward normal. It completely abolishes the rat’s interest in cats and restores their normal fear (Prandovszky et al., 2011).

It may be that while there are many individuals in the addictive drug industry who consciously manipulate the brains of their customers, there are other individuals such as physicians who are mystified by the way their attempts to help patients with pain or anxiety using medications in the opioid and benzodiazepine classes results in addictive behaviors. Patients who were initially grateful for the help of the physician later begin to manifest manipulative and hostile drug-seeking behaviors that cause consternation in the physician. It may be that without thinking about this process, physicians are seeding their patient population with medications that may become urgently wanted by some who undergo the brain change described here. This formulation about how drugs alter the will may facilitate more careful prescribing.

## SUMMARY

Combining developing concepts about the brain effects of addictive drugs with psychoanalytic observations, new hypotheses about the disease of addiction have been generated. Addictive drugs take over the will by transiently increasing dopamine firing in the SEEKING pathway. A new drive to obtain the drug results in the formation of a series of psychological defenses that both promote gratification and shield the person from the anxiety produced by addictive behaviors, the denial system. Idealization of the drug is a ubiquitous defense. Addicted persons cathect the seller of the drug as a result of repeated gratification of the drive. They fall in love with the drug and the seller/dealer.

The first task in treating actively addicted patients involves negotiating the split cathexis. The treater rides the line between ignoring the addiction, and directly opposing the cathexis with the drug/drug dealer. Clarification and confrontation are the two main types of interpretation that are used until alcohol or drug use ceases.

After cessation of use, the treater has to explore underlying hostile/aggressive urges and dependency needs that had been encapsulated by the addictive behavior. It is rare to see a purely physical addiction. In most cases, the very reason that the addiction was adopted has to do with an inability to use aggression effectively to negotiate relationships and inability to depend on people. Without a thorough exploration of these dynamics, the patient is prone to relapse to use of the addictive drug.

Selling addictive drugs is a huge industry in the world. Sellers use their understanding of the psychodynamics of addiction to capture brains. A public health approach can use the formulations above to help potential victims understand that the drugs work by taking over the will and causing warm feelings toward individuals who don’t mind having their customers die.

The author has suggested a homology between the way the *T. gondii* parasite takes over the will of the rat, and the way addictive drugs take over the will of a person. In both instances “the will” involves dopaminergic function. One aspect of the behavior of the victim involves being willing to sacrifice their life to propitiate the welfare of the parasite or the welfare of the drug dealer.

## Conflict of Interest Statement

The author declares that the research was conducted in the absence of any commercial or financial relationships that could be construed as a potential conflict of interest.
